# Supplementary figures and images for: Influence of previous Zika virus infection on acute dengue episode
Source: PLoS Negl Trop Dis. 2023 Nov 9;17(11):e0011710. doi: 10.1371/journal.pntd.0011710 (PMC10662752; doi:10.1371/journal.pntd.0011710)

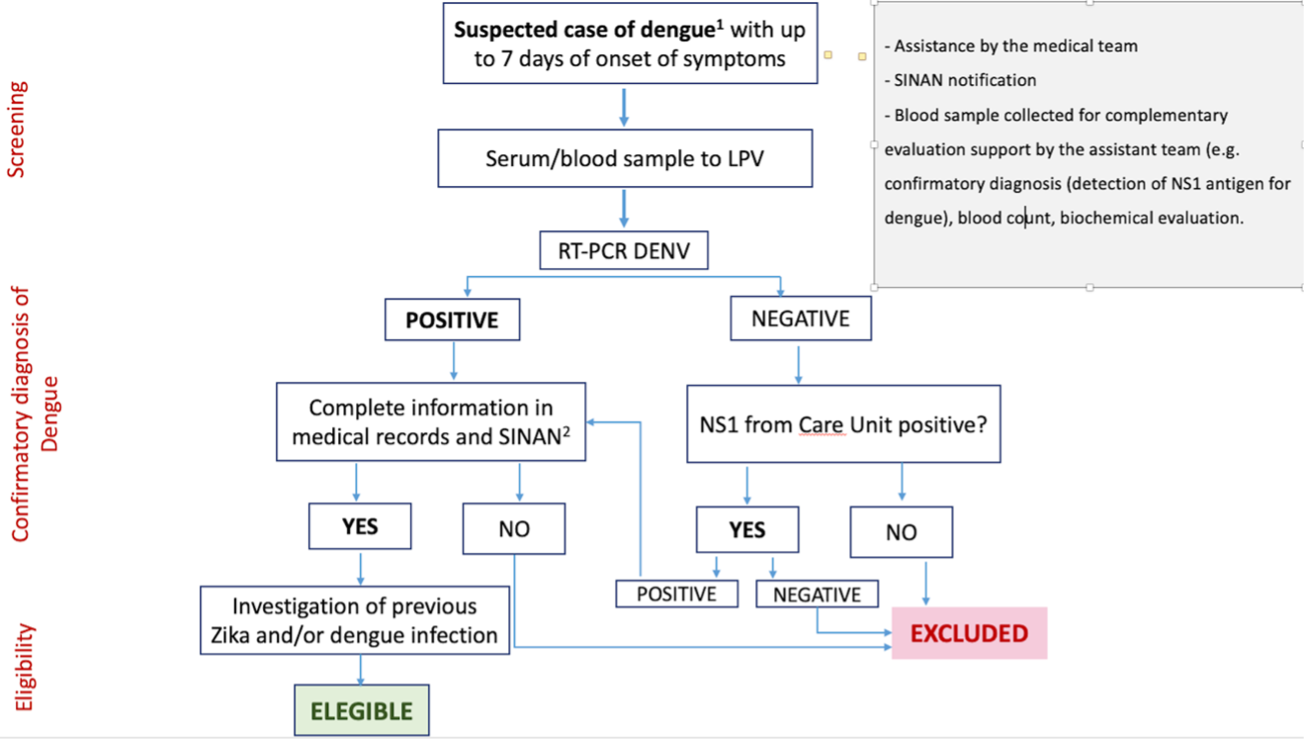

Supplement: S1 Fig — (TIF) [file pntd.0011710.s001.tif]

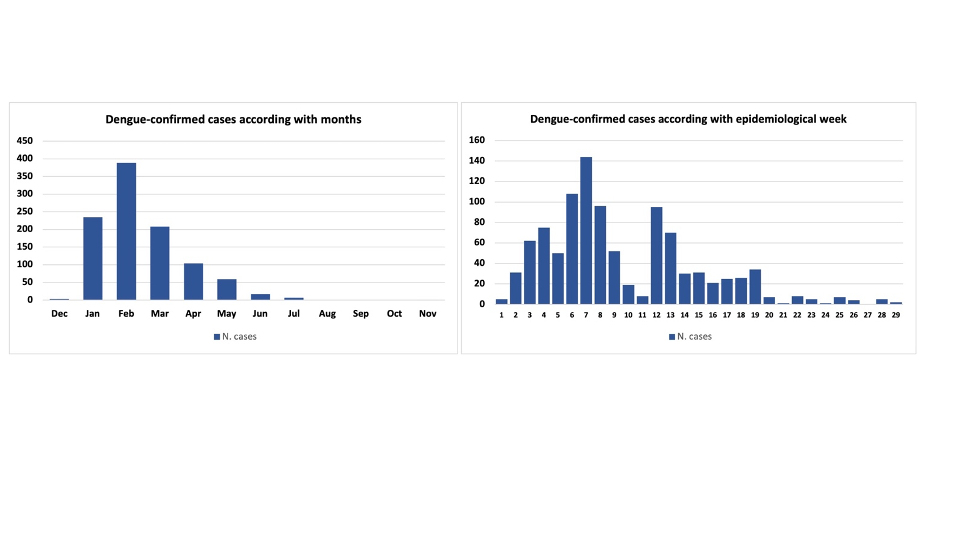

Supplement: S2 Fig — Receiver Operating Characteristic (ROC) curves of pepELISA for the peptides (A) DV-15; (B) DV-20; (C) ZV-54; and standard ELISA against (D) DENV NS1 mix, and (E) ZIKV NS1. ‘Control’ identifies samples considered true negatives, and ‘Patients’ identifies samples considered true positives. Performance is demonstrated in True Positive Rate (Sensitivity %) versus False Positive Rate (100%—Specificity %). (TIF) [file pntd.0011710.s002.tif]
