# Supplementary material for: Influence of previous Zika virus infection on acute dengue episode
Source: PLoS Negl Trop Dis. 2023 Nov 9;17(11):e0011710. doi: 10.1371/journal.pntd.0011710 (PMC10662752; doi:10.1371/journal.pntd.0011710)
Supplement: Attachment — Submitted filename: response to reviewers.docx [file pntd.0011710.s008.docx]

São José do Rio Preto, 28^th^ August 2023

Shaden Kamhawi, Ph.D.

Editor-in-Chief

*PLOS Neglected Tropical Diseases*

Ref: PNTD-D-23-00392R1.

Dear Dr. Kamhawi,

Thank you for your consideration of our manuscript. On behalf of all authors, we appreciate the reviewer’s constructive comments of our work. We have now addressed all the reviewers’ concerns and all changes are highlighted in colored text as outlined below.

Best regards,

Mauricio Lacerda Nogueira, MD, PhD

Faculdade de Medicina de São Jose do Rio Preto

São Jose do Rio Preto, SP, Brazil

University of Texas Medical Branch

Galveston, TX, Brazil

***Point-by-point Responses to Reviewers Considerations***

***1. Editorial and Data Presentation Modifications***

*Reviewer #3*

*In this paper the authors looked at the flaviviruses’ (zika & dengue) immunity background in 1,043 laboratory confirmed dengue patients. The finding was that prior zika is a risk factor for severe dengue, even more than prior dengue. I find this observation extremely important as the entire world is occupied with the sequence of second dengue post dengue infection. If this observation is found to be true in subsequent studies, then the concept of severe flavivirus infection should be revised to include prior other flavi or flavi viruses’ vaccines as a risk factor and not just various dengue serotypes.*

*The other interesting point is the importance that the authors gave to look for other explanations for severe disease rather than the common theory of ADE.*

*The basis for the observation of this study is the authors’ ability to identify accurately previous flaviviruses infection. They mentioned that they developed and validated a new peptide-based Enzyme-linked Immunosorbent Assay (ELISA) method for dengue and Zika detection, which I am not familiar with. I think they should elaborate more on this method.*

**Response:** We appreciate the reviewer 3's comments in recognizing our efforts to expand the understanding of severe forms of dengue. Although ADE is classically associated to the development of severe dengue, there has been an increasing frequency of observations that other factors are involved, including comorbidities and, as demonstrated in this study, prior Zika infection. Indeed, this correlation can also be supported by data published by Katzelnick et al (2020) [doi: 10.1126/science.sbb6143].

In regard to the PepELISA method, we would like to draw attention to the submitted supplementary material that contains a detailed description of its development and validation. Briefly, we used previously described monoclonal antibody pairs specifically designed to recognize NS1 protein to DENV and ZIKV. The application of these antibody pairs as a diagnostic tool was presented by Bosch et al (2020) [doi: 10.1371/journal.pntd.0008203]. We used these antibodies to select NS1 peptides from an epitope library based on binding affinity to the specific DENV or ZIKV pairs. Later on, the selected peptides were tested against a validated sera bank (monotypic PRNT-confirmed serum samples), where peptides were immobilized at the surface of PVC plates – as previously published by our group (Versiani et al, 2019) [doi:

10.3390/v11111079]. Moreover, here we included a broader description of the assay on page 9, lines 196-200, as “For this, DENV1-4 and ZIKV NS1 libraries of 13-to 19-mers peptides, with variable amino acid overlaps, were tested against DENV and ZIKV specific monoclonal antibodies pairs described elsewhere. After selection, the Peptide ELISA IgG assay (PepELISA) was analyzed with samples serologically validated by plaque reduction neutralization test (PRNT).”, in addition to the detailed methodology in the main text due to both reviewers’ requests for additional information regarding this assay.

*Minor comments*

*Abstract: 
Background: 
L. 38 "The simultaneous circulation of flaviviruses…" I prefer to say the co-circulation.*

**Response:** Corrected as suggested.

*L.  39 "the hypothesis that immunity generated by a previous flavivirus could promote severe disease outcomes in subsequent infections by heterologous flavivirus". The accepted theory until now is in regard to heterologous dengue serotypes and not among all flaviviruses.*

**Response:** The text was corrected as follows: “…the hypothesis that immunity generated by a previous dengue infection could promote severe disease outcomes in subsequent infections by heterologous serotypes”.

*Introduction
L. 70-" Infection by any of the four DENV serotypes causes acute febrile illness" –this is not true as most cases are probably asymptomatic.*

**Response:** Corrected as suggested

*L. 87 –" protocols in treating exacerbated disease outcomes" – the wording is not clear.*

**Response:** The text was corrected to “Therefore, understanding the role of pre-existing immunity to genetically and antigenically similar pathogens may lead to effective clinical protocols to treat or control exacerbated forms of the disease.

***2. Summary and General Comments***

*Reviewer #1*

*Immune system interactions between closely-related flaviviruses during sequential infections can modulate an individual’s response to subsequent flavivirus infections. This is best exemplified in the setting of dengue virus, where pre-existing immunity to one dengue virus serotype can be protective, be neutral, or can enhance the risk of symptomatic disease during secondary infection, depending on the order in which those serotypes were encountered. In addition, emerging evidence from humans suggests that pre-existing immunity to Zika virus can enhance the risk of subsequent dengue virus infection. Here, Estofolete et al. use human samples collected from a region in Brazil that is hyperendemic for dengue virus but also previously experienced Zika virus circulation during the 2015-16 outbreak, to explore the role of pre-existing Zika virus immunity on subsequent dengue virus infection outcomes. They further evaluate the role that antibody-dependent enhancement might play in mediating the phenomena that they observe. Overall, this is a unique study with some unique aspects and thus an important contribution to the literature. However, I believe there are a few scientific issues and several things that need to be addressed to facilitate understanding by the reader. First, grammar and syntax need to be improved throughout, and the manuscript should be revised for clarity. In several instances it was difficult to follow exactly what was done with each sample. For example, a major component of this study is the development of an ELISA assay that can differentiate past ZIKV from DENV infections and vice versa, but it is not completely clear if this is simply proof-of-concept with a subset of samples or how this assay contributed in a significant way to delineating the 1,043 samples that were used for the analyses presented herein.*

*In addition, more details should be presented on the dengue virus serotypes that individuals were infected with both as part of this study and in the past. If this information is not known that is okay, but some language should be included at the outset that describes how the analysis will focus on outcomes in a serotype-independent manner then.*

**Response:** We thank reviewer 1 for appreciating the findings of this study and its significance in the global landscape of arbovirus infections. We take this opportunity to better explain the samples' selection criteria and the assay we developed to investigate previous infections (PepELISA IgG). The selection criteria for inclusion of samples are presented in the supplementary figure S2. For developing and validating the peptide ELISA assay, we utilized a subset of 170 samples obtained from 2006 to 2017 and previously serologically validated (ELISA and PRNT) from follow-up of acute patients confirmed by RT-PCR (in the case of Zika patients). The detailed methodology of development and validation of the PepELISA was included in the main text. After validation of the assay using characterized samples (Figures 1, 2, and 3, and Table 1), we tested archived samples screened for dengue in the 2019 outbreak to identify previous infections of dengue and Zika (Figure S2). The presence of IgG antibodies against ZIKV and/or DENV was utilized as the basis for the following statistical analysis of the hospital cohort (1,043 samples).. Regarding to the circulating serotypes, it was laboratory confirmed that 99.8% of the identified dengue infections were caused by DENV-2. This data has been included in **Results section, General characteristics of the study population sub-section**, as “DENV-2 was the most frequent serotype identified (99.8%).”

*Other specific comments follow:*

*Line 89: does it really matter if ZIKV emerges in areas where dengue is hyperendemic? If immune system interactions between DENV and ZIKV really matter, I would think that there is a concern about co-circulation of these two viruses wherever they are endemic.*

**Response:** We fully agree with the reviewer. The term "hyperendemic" was used to accurately depict the area where our study was conducted, but its significance applies to all areas where both viruses are endemic. Therefore, the text has been corrected to *“*in areas of endemic ZIKV and DENV circulation”.

*Line 91: the transition to this paragraph is awkward and confusing.*

**Response:** We thank the reviewer for the suggestion. Corrected as suggested.

*Line 100-102: this should not be a stand alone paragraph and should be incorporated into the paragraph above describing problems associated with serological diagnosis of these two viruses.*

**Response:** *Corrected as suggested.*

*Line 103-120: this section of the introduction should be improved since it represents the underlying premise of the study.*

**Response:** The introduction section was rewritten, and more aspects to the design and background were included.

*Line 304-305: I respectfully disagree with the statement that both DENV peptides efficiently separate DENV-positive from ZIKV-positive individuals. DV20 looks the best in this regard, but both DV20 and DV15 show some cross-reactivity with ZIKV-positive individuals—with DV15 showing significant cross-reactivity. All 3 peptides suffer from false negatives and false positives. In reality, it is probably unreasonable to expect these to be 100% and 100% specific but some context on relative performance to other assays would be appreciated here to understand how useful this tool is for the studies described here and for the field at large.*

**Response:** We fully agree with the reviewer and we have re-evaluated how to present the diagnostic performance of the assay in a more relevant way. As described in our data (Figure 3 and Table 1), both selected peptides – especially DV20 and ZV54, present a better ROC curve and diagnostic performance parameters (accuracy, sensitivity, and specificity) in comparison with the DENV/ZIKV NS1 protein tested with the same samples. An important reminder for this analysis is that the ROC curve and sensitivity/specificity analysis are classically established to evaluate the accuracy of an assay against true-positive (DENV+ sera or ZIKV+ sera) and true-negative samples (NEG sera). In this regard, peptide DV20 presented a sensitivity of 95.7% and specificity of 94.6%, consistent with other well-validated antigens commercially applied as diagnostic tools. However, this analysis only reveals the ability of the peptide to separate true positives from true negatives – and does not truly compare the serological detection among viruses.

Taking into consideration Reviewer 2 interpretation of this data, we re-run the analysis in a non-classical way, including ZIKV positive sera into the true negative category and analyzing the diagnostic performance of the DENV peptides and protein. The opposite was performed for ZIKV. In this scenario, we are effectively comparing DENV to ZIKV sera diagnostic performance. Observing the new ROC curves obtained from this analysis (Fig S3), both NS1 proteins perform poorly – with specificity varying from 56% to 70% among them (Table S1). The peptides, conversely, can clearly identify DENV or ZIKV more accurately than the standard assay containing the complete NS1 protein.

*Line 325: how were past DENV and ZIKV infections confirmed in the patient's medical records? Are these only individuals with a molecularly confirmed or NS1 antigen test? Or do these samples include individuals that had infections confirmed by serology? For those with past DENV infections, is it possible to know how many and the serotype?*

**Response:** Previous infections of dengue or Zika were not checked or confirmed in the medical records. They were determined by our developed ELISA method. The text was updated appropriately.

*Line 329-331: This section is a little confusing. It would be helpful to clarify that these specimens are from individuals in which the DENV infection could be their 1st, 2nd, or 3rd infection in a series of flavivirus exposures.*

**Response:** Unfortunately, we do not have this information, since only ELISA was performed to identify prior infection to Zika or dengue. We recognize that PRNT would be the ideal tool to confirm, but unfortunately there are no remaining samples to be tested again.  *Line 364: please add the sample size for this group with severe bleeding.*

**Response:** This data is presented in Table 3. We had 3 severe bleeding cases, 2 in DV-/ZV+, 1 in DV+/ZV-, and 0 in DV-/ZV-. The data was added to text as “2/31 (6.45%) in DV-/ZV+ vs. 0% in control”.

*Line 362-372: these analyses are for all dengue in general, but is there information on serotype, and if there is can you do more fine-scale analysis to understand serotype-specific interactions between DENV and ZIKV outcomes?*

**Response:** Correct. There is information about the dengue serotype was added in the Results section, that serotype 2 was predominant in the 2019 dengue epidemic in our study area, accounting for 99.8% of all cases. Therefore, it is not possible to conduct an analysis based on serotype, given the high frequency of only one of them.

*Line 370: I don’t understand the comparison listed here. The 2 outcomes used were resolution of infection and death. Death does not seem like an appropriate outcome to use for these types of analyses. I would suggest re-doing analyses with symptomatic infection and/or severe symptoms as the outcome.*

**Response:** The two outcomes used in our analysis were resolution (cure) and death, which was performed and presented in Table 3 as DwWS (dengue without warning signs) versus DWS + SDD (severe forms of dengue, being DWS = dengue with warning signs and SDD = severe dengue disease). These findings are included in lines 366-367 as “When the cases were clinically classified, there was a higher occurrence of severe and potentially severe forms (SDD+DWS) in the DV-/ZV+ group (p<0.001).”

*Discussion: the discussion is overly long and could be tightened up to focus on how this study is or is not consistent with the existing literature, potential mechanisms that might underlie the interaction, and where the field might go from here.*

**Response:** The discussion section was rewritten, with a focus on the consistencies and inconsistencies in literature, including caveats/limitations of our study and our indication in sharing our data with the scientific community

Reviewer #2

Novel study showing the casual relation of zika and Dengue severity but need to find evidence of antibody-dependent enhancement can be emphasized.

**Response:** We respectfully disagree with the Reviewer on this as antibody dependent enhancement (ADE) disease model for dengue, is not the focus of our manuscript. Our study’s focus is based on the influence of Zika infections on subsequent dengue infection. Our data suggest a protective effect of previous dengue (Table 4, logistic regression; p<0.001) and do not correlate to secondary dengue, whose ADE is characterized, and cited a manuscript that shows our study area is hyper endemic for arbovirus transmission and its population has been repeatedly exposed to several heterologous DENV. In addition, our findings in DV-/ZV+ samples do not correlate to ADE (high viral load, cytokine expression profiles).

Reviewer #3

The basis for the observation of this study is the authors’ ability to identify accurately previous flaviviruses infection. They mentioned that they developed and validated a new peptide-based Enzyme-linked Immunosorbent Assay (ELISA) method for dengue and Zika detection, which I am not familiar with. I think they should elaborate more on this method.

**Response:** We thank the reviewer for the opportunity to provide further details on the development and validation of the peptide-based ELISA. The complete methodology was added in the main manuscript, with a new differential analysis of the diagnostic performance of the pepELISA in comparison to the standard assay performed with the whole antigen (NS1).
